# Review of the Palaearctic *Acomopterella* Zaitzev (Diptera, Sciaroidea, Mycetophilidae)

**DOI:** 10.3897/zookeys.269.4252

**Published:** 2013-02-15

**Authors:** Uwe Kallweit

**Affiliations:** 1 Senckenberg Naturhistorische Sammlungen Dresden, Museum für Tierkunde, Königsbrücker Landstrasse 159, D-01109 Dresden, Germany

**Keywords:** Taxonomy, Sciaroidea, Mycetophilidae, *Acomopterella*, new species, Central Europe, North Europe, Alps, Sakhalin, Hokkaido, Honshu, morphology, functional specialized setae

## Abstract

The distribution of *Acomopterella* species in the Palaearctic region has been re-examined in this study, using recently collected material. The European species was found to be distributed in the eastern Palaearctic as well. A second Palaearctic species from Honshu (Japan) is herein described. The morphology of adult specimens was studied by light microscopy and scanning electron microscopy. The shape of functional specialized setae on mid tibiae in *Acomopterella* and seven further fungus gnat genera is described and the suitability of this character for systematic studies is discussed. Details of a “hind tibial organ” are described.

The position of *Acomopterella* in the tribe Gnoristini is briefly discussed. *Acomopterella* is found to be more closely related to *Speolepta* Edwards, 1925, than to any other recent genus.

## Introduction

Sciaroidea, i.e. fungus gnats s.l. are of global distribution, quite diverse, with far more than 10.000 named species. Fungus gnats are excellent indicators in the assessment of forest habitats in terms of nature conservation. Mycetophilidae and Sciaridae play an important role in the economy of nature, even if that has not yet been shown by quantitative analyses. Several ecological and faunistic studies give an impression of the predominance of this Diptera group, especially in woodland habitats, e.g. Engel (1995), Kleinevoss et al. (1996), Heller (1996). The number of described species is constantly increasing, and the identification of single species is becoming progressively safer and easier. The present study provides new taxonomic and distributional data on the genus *Acomopterella* Zaitzev, which was defined in 1989, for a species from the Western Nearctic. The second species of this genus, *Acomopterella martinovskyi* Ševčík & Chandler, 2008 was described from several places in the Czech Republic and from the Tyrolean Alps. The authors also synonymised the type species of the genus with a species previously described in the genus *Tetragoneura* Winnertz, 1846, a global genus with a vast number of named species. The present investigation has led to additional data and discovery of an undescribed species.

## Material and methods

This study is based primarily on material from the Senckenberg Naturhistorische Sammlungen Dresden (SNSD), partially collected by the author. Malaise trap and sweep net samples from the Bavarian Alps, and from the islands of Sakhalin and Honshu were included. The type specimen of the newly described species is deposited in Dresden.

In addition to the two species treated in detail below, one specimen of *Archaeboletina tipuliformis* Meunier, 1904, a Baltic amber fossil was studied. Besides the species description, a second focus was directed on the ultrastructure of functional specialized tibial setae in 13 Mycetophilidae species. Most previous studies have been carried out using light microscopy; the present one was performed with scanning electron microscopy (SEM). In many cases optical refraction does not allow the user to study details of hyaline structures, but SEM does. Functional specialized tibial setae were studied in *Acomopterella martinovskyi*, *Acomopterella yoshiwae* sp. n., *Speolepta leptogaster* Winnertz, 1863, *Ectrepesthoneura hirta* Winnertz, 1846, *Tetragoneura* (2 unidentified species from the Far East and from New Zealand), *Synapha fasciata* Meigen, 1818,* Docosia diutina* Plassmann, 1996, *Coelophthinia thoracica* Winnertz, 1863,* Phthinia humilis* Winnertz, 1863,* Phthinia winnertzi* Mik, 1869,* Polylepta guttiventris* (Zetterstedt, 1852) and *Polylepta borealis* Lundström, 1912.

Specimens are mounted on microscope slides or kept in 70% ethanol. The amber fossil has been preserved in polyester, following Hoffeins’ preparation method (2001). They were studied with the aid of an Olympus SZH10 stereomicroscope. For light microscope study and the preparation of drawings an Olympus BH2 was used. Photographs were taken with a digital Olympus C-3030 camera attached to Olympus SZH10 and BH-2 microscopes. SEM photos were taken by a Zeiss SmartSEM^TM^, Type Supra 55VP. Length measurements were taken of slide-mounted specimens only. The lengths of wing veins were assessed with the base of the stem vein as the basalmost point. The wing index, used to describe the shape of the wing, is the ratio of the wing length to its width. Morphological terminology, including abbreviations, follows McAlpine (1981), Søli (1997), Matile (1990) and Blaschke-Berthold (1994). The terminology in the egg description follows Mazzini et al. (1992) and Plachter (1981). The genus definition, given by Zaitzev (1989), has been adopted. *Acomopterella martinovskyi* is re-described at length, based on eastern Palaearctic specimens.

## Taxonomy

### Genus Acomopterella

Zaitzev, 1989

http://species-id.net/wiki/Acomopterella

Acomopterella Zaitzev, 1989: 134 (description); Ševčík & Chandler: 2008: 63 (discussion).

#### Type species.

*Acomopterella arnaudi* Zaitzev, 1989: 134 (by original designation) = *Tetragoneura fallax* Sherman, 1925: 20.

#### Diagnosis.

(based on Zaitzev’s original genus description, with additions).

**Body** length 4.6-5.9 mm. **Head**. With 3 ocelli. Mid ocellus slightly in front of the lateral ocelli. Lateral ocelli scarcely to distinctly remote from eye margin. Eyes with short setulae. Face rather flat, bare or setose. Clypeus oval, slightly bulging, setose. Face and clypeus loosely connected. Palpi pentamerous. Antennae 16-segmented, 2-3 times length of the thorax. Length of mesial flagellomeres 3-5 times their own width. **Thorax**. Scutum with short, close lying acrostichal and long erect dorsocentral bristles. Scutellum with 2 very long medial bristles. Mediotergite, laterotergites and pleura bare. **Wing**. Transparent. Wing membrane with microtrichia only. C, the apical part of ta, R1, R5, M1, M2, M4 and CuA with macrotrichia. C extending to the tip of R5. R4 present; ta oblique, slightly shorter than stem of fork M1 + M2. Base of M4 on level of base of ta or slightly more proximal. Vein A well developed, its tip behind the level of base of ta or ending slightly before. Between A and the stem of fork M4 + CuA lies the weakly sclerotized vein CuP, traceable as fold line. **Legs.** Long. Fore coxae with numerous long bristles on its frontal side. Apical part of mid coxae with bristles on its lateral and frontal surface. Hind coxae with a row of very long bristles on the hind lateral surface. Mid tibiae with well developed tibial organ on its basal part. Tarsal claws with teeth. Empodia well developed. **Abdomen**. Segments 1–7 of normal size. Tergite 8 vestigial; Sternite 8 exceeding half length of sternite 7. Tergite 9 rectangular, with deep distal incision. Gonocoxites with apical processes.

#### Comments.

The described genus belongs to the tribe Gnoristini in the subfamily Sciophilinae. It is similar to *Dziedzickia marginata* (Dziedzicki 1885), but differing by bare laterotergites and a much longer vein ta. It differs from the Holarctic genus *Acomoptera* Vockeroth, 1980, in the position of Sc (in *Acomopterella* it joins R1, in *Acomoptera* C), in the shape of the cell between Rs and R4 (distinctly longer in *Acomoptera*) and in the strikingly longer ta. The genus is also similar to *Austrosynapha* Tonnoir, 1929, known from South America, New Zealand and Tasmania. Typical in both genera is the absence of setae on the laterotergites and the presence of a relatively long oblique vein ta. In *Austrosynapha* vein R4 is absent.

### Acomopterella martinovskyi

Ševčík & Chandler, 2008

http://species-id.net/wiki/Acomopterella_martinovskyi

**Figures 1–8.**
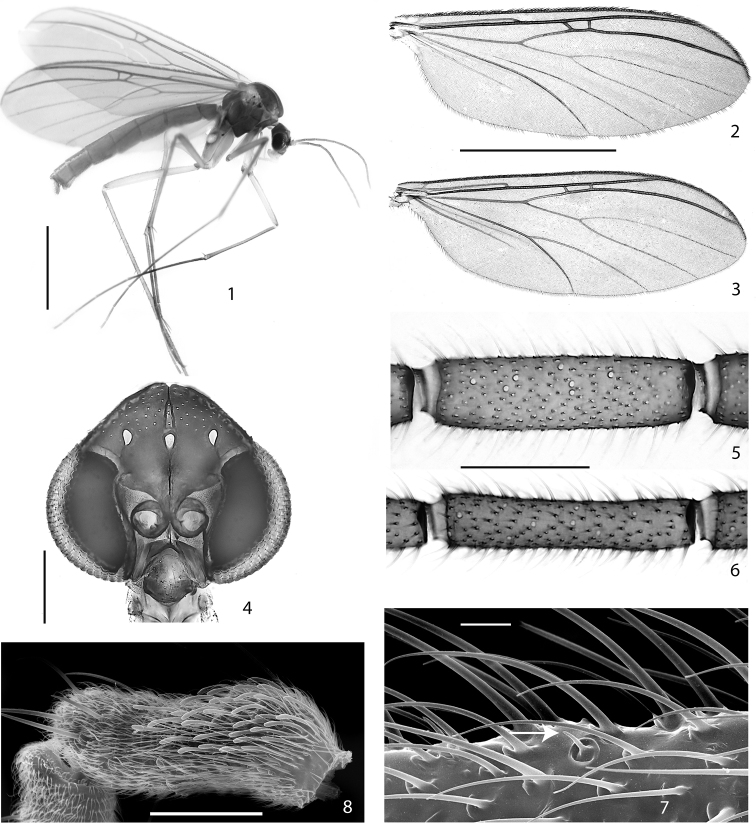
**1–2, 4–5, 7–8.** *Acomopterella martinovskyi*. **1** Female habitus **2** Male wing **4** Male head, frontal view **5** Flagellomere 4, male. Sensilla chaetica visible as pale spots. **7** Sensillum chaeticum (at arrowhead) on flagellomere 10, male **8** Sensilla on palpomere 3, male **3, 6** *Acomopterella yoshiwae*sp. n., male. **3** Wing **6** Flagellomere 4. Length of scale bar = 2 mm (for **1–3**), 200 µm (for **4**), 100 µm (for **5–6**), 10 µm (for **7**), 50 µm (for **8**).

Figs 1–2, 4–5, 7–8 10–13 14–20 33–34

#### Diagnosis.

A distinctive species with vein M4 basally detached, thus no complete hind fork present (Fig. 2). There is a tendency to basalization of radial veins, so that R1 is foreshortened. C extending beyond apex of R5 for 1/3 distance to M1. Subcosta ending directly in R at the level of base of ta. Crossvein ta nearly horizontal, long, subequal in length to M-stem. A single setum chaeticum of the mid tibial organ has 7-9 filamentous branches (Fig. 34). Empodium well developed (Fig. 12). Gonostylus simple, tapered (Fig. 14). Gonocoxites ventrally widely separated, having only a narrow basal sclerotized connection. Cerci with strikingly dense, long hairs, which are mostly directed proximad (Figs 18–20).

#### Description.

Male. **Head.** Fig. 4. Head capsule, labella and antennae brown, palpi yellow. With three ocelli in a broad triangle. Median ocellus slightly smaller than the lateral ones. The median ocellus is surrounded by the medioocellar suture, with narrow pseudosclerite above ocellus. Lateral ocelli less than two times its own diameter distant from eye margin. Compound eyes covered with interommatidial setulae, with only some few blank interommatidial spaces near the dorsal eye margin. Ommatidia densely arranged. Basal palpomere distinct, ventrally extended, covering palpomere 2 for some distance. Second palpomere quite small, with 1–2 minute setae. Third palpomere with numerous sensilla claviformis that are evenly dispersed over the basal 3/4 of the inner surface of palpomere 3 (Fig. 8), a few setae situated mainly apically. Palpomeres 4 and 5 loosely scattered with similar setae, as present in the preceding segment. Scape and pedicel with few stronger setae, flagellomeres 1–3 with few small setae dorsally. Flagellomeres cylindrical, with trichia not reaching the length of a flagellomere diameter (Fig. 5). The antennal flagellomeres are sparsely scattered with sensilla chaetica (Figs 5, 7), with a decreasing number of sensilla towards the tip of the flagellum. The antennal surface between the described vestiture is smooth. Antenna reaching 4^th^ abdominal segment. Face with 4–6, clypeus with several more setae. The premental apodeme is small, its posterior part weakly sclerotized and hardly traceable.

**Thorax.** Fig. 10. Uniformly brown. Scutum shining, dome shaped, with long erect acrostichal, dorsocentral and lateral setae. Antepronotum with 2 large and a further few tiny setae, proepisternum with 1-2 large setae and a few tiny setae. Meso- and metapleuron, laterotergite and mediotergite bare. Scutellum with 2 strong and several smaller setae.

**Wing.** Fig. 2. Length 3.8-4.7 mm. Wing index 2.6. Wings clear, membrane with microtrichia only. Subcosta ending at level of base of ta in R, or shorter. Radial veins, M1, M2, M4 and CuA setose on the dorsal surface, R1 ventrally setose at tip. Other veins bare. Stem of median fork long, about 0.16 times wing length. The position of vein R4 is slightly variable, depending its distance from Rs. Vein R5 a little downcurved apically, costa exceeding its tip for one third of its distance to M1. M4 long, visible far before level of ta-base, basally detached. Haltere yellow.

**Legs**. Entirely pale brown. Hind coxae on its posterior ridge with a row of setae. Tibial and tarsal trichiae irregularly arranged. Hind tibiae with 4 ventral, 3 posterior, 2 posterodorsal, 1 dorsal, 6-7 anterodorsal and 10 anterior setae, the posterodorsals as long as tibial width, the other are shorter than tibial width. Mid tibia with 3 ventral, 3 anterior, 3 dorsal and 5-7 posterior setae, all of them shorter than tibial width. Fore tibia with 3 dorsal and 4 posterior setae. Mid tibia slightly swollen at its base, with an elongated patch of specialized seta chaetica (Figs 33-34). The single setum chaeticum has 7-9 filamentous branches, subequal in length and width. Hind tibia without apical comb. Tibial spurs 1:2:2, mid and hind spurs longer than tibial width. Empodium of normal size (Fig. 12). Fore basitarsus 1.1 times longer than tibia.

**Abdomen**. All abdominal segments including terminalia brown, with pale hairs. Segments 2–5 long, 6 and 7 a little shorter. Tergite 8 vestigial (Fig. 17) and completely retracted, sternite 8 reduced in width, partly retracted. **Terminalia.** Figs 14–20. Tergite 9 medium sized, twice as broad as long, together with cerci covering 3/4 of the gonocoxites. Gonocoxites ventrally widely separated, having only a narrow basal sclerotized connection. Parameres long, hardly reaching gonocoxal apex. Gonostylus medium sized, about half as long as gonocoxite, very slightly (Europe) to double bent (Far East) and simply tapered. European specimens have the gonostylus slightly shorter, maybe reaching 1/3 of gonocoxite length. Cerci with strikingly dense, long hairs, which are directed proximad. Hypoproct weakly sclerotized, horseshoe-shaped, with two strong apical setae (Fig. 16).

Female. **Head**. Antenna reaching 3^rd^ abdominal segment. **Legs.** Front tarsi unmodified, except for the prolonged basitarsus. No mid tibial organ present. **Terminalia.** Gonocoxite 8 widely rounded, with a couple of distad directed apical setae (Fig. 13). Tergites 9 & 10 sparsely setose. Cerci setose, basicercus twice as long as disticercus. Cerci directed ventrad. **Egg**. A single egg is 410 µm long and 135 µm in diameter. The dorsal and lateral parts of the eggshell are characterized by indistinct longitudinal rows of rosette-like, hexagonally arranged superficial layers, reaching the posterior pole (Fig. 21). The dorsal and lateral surface is described best as being irregularly rough and sharp-edged (Fig. 25). The micropyle is distinct, its surrounding area with 10-20 isolated plugs (Fig. 22). The lateral transition zone has the hexagonal structures gradually suppressed. The ventral surface is more flattened and rugose (Fig. 21), with a central plate of differing structure. The ventral plate has a rounded outline and is distinguished by dense-set, simple to three-pieced upright columns (Figs 23, 24). The egg of *Acomopterella martinovskyi* is distinct from that of other fungus gnat species by its rough dorsal and lateral surface. It resembles other species in the general structure of the micropylar area, the flattened bottom and the presence of a ventral plate. The structure of a ventral plate on Mycetophilidae eggshells was described by Mazzini et al. (1992, p. 34) for the first time, in 3 *Mycomya* species.

#### Distribution and phenology.

*Acomopterella martinovskyi* is known from several localities in the Czech Republic, from the Tyrolean Alps (Austria), the Bavarian Alps (Germany), from Sweden (Kjaerandsen 2012), from Sakhalin (Russia) and Hokkaido (Japan).

#### Material studied.

2 males (on slide and in ethanol), Germany, Bavaria, Allgaeu, Mt. Ponten (Alps), East of Hinterstein, SE of Sonthofen, 1840 m, 47°31'N, 10°18'E; Malaise trap; collector Voith. 1 male (in ethanol), Russia, Sakhalin Island, southern part near city Yuzhno Sakhalinsk, 200 m; mixed broadleaved forest; 142°45'E, 47°00'N ;1–5 Oct. 1993 Yellow pan trap; 200m; collector U. Kallweit. 1 female, 4 males (on slide), Japan, Hokkaido, Ogusawa-suigenchi area; near city of Otaru; 141°00'30"E, 43°10'30"N, 100m; 28 June 1997; sweep net, collector U. Kallweit.

Other material, pictures taken by electron-scanning microscope: 1 male. Japan, Hokkaido, Ogusawa-suigenchi area; near city of Otaru; 141°00'30"E, 43°10'30"N, 100m; 28 June 1997; sweep net, collector U. Kallweit.

### Acomopterella yoshiwae

Kallweit sp.n.

urn:lsid:zoobank.org:act:1C2B125A-45E0-453D-9124-4E673CEAC095

http://species-id.net/wiki/Acomopterella_yoshiwae

Figs 3, 6 , 9 , 26–30 , 31–32

#### Diagnosis.

The species is characterized by a peculiar extended fore basistarsus, which is 1.5 times as long as the fore tibia. A strong basalization of radial veins has led to the nearly horizontal crossvein ta and foreshortened R1. C extending beyond apex of R5 for 1/3 distance to M1. Subcosta ending a short distance before base of ta in R. Crossvein ta not reaching the length of M-stem. A single setum chaeticum of the mid tibial organ has 2 filamentous branches (Fig. 32). Empodium well developed. The gonostylus of *Acomopterella yoshiwae* consists of two completely separated lobes (Figs 26–27). Gonocoxites ventrally separated, having only a narrow basal sclerotized connection. Cerci small, with few apical setae (Fig. 28).

#### Description.

Male. **Head.** Head capsule, labella and antennae brown, palpi yellow. With three ocelli in a broad triangle. Median ocellus half the diameter of the lateral ones. Median ocellus surrounded by the medioocellar suture, with a narrow pseudosclerite above the ocellus. Lateral ocelli nearly contiguous to eye margin. Compound eyes covered with interommatidial setulae, with only some few blank interommatidial spaces near the dorsal eye margin. Ommatidia densely arranged. Palpi as in *Acomopterella martinovskyi*. Scape and pedicel with few stronger setae, flagellomeres without setae. Flagellomeres cylindrical, with trichia not reaching the length of a flagellomere diameter (Fig. 6). The antennal flagellomeres are sparsely scattered with sensilla chaetica (Fig. 6), with a decreasing number of sensilla towards the tip of the flagellum. The antennal surface between the described vestiture is smooth. Antenna reaching 4^th^ abdominal segment. Face and clypeus far-reaching fused, with few setae. The premental apodeme is small, posterior part weakly sclerotized and hardly traceable.

**Figures 9–13.**
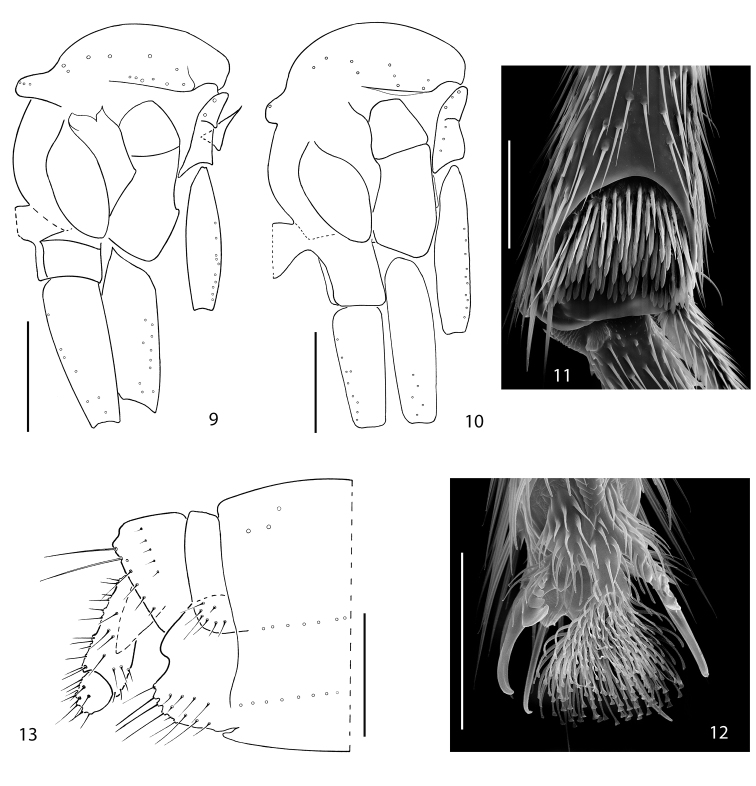
**9** *Acomopterella yoshiwae*sp. n., thorax in lateral view. **10–13** *Acomopterella martinovskyi*. **10** Thorax in lateral view **11** Fore tibial organ, male **12** Fore claw, male **13** Female terminalia in lateral view. Length of scale bar = 0,5 mm (for **9–10**), 50 µm (for **11–12**), 250 µm (for **13**).

**Thorax.** Fig. 9. Uniformly brown. Scutum rather flattened; with pale, long erect acrostichal, dorsocentral and lateral setae. Antepronotum with 2 large and a further few tiny setae, proepisternum with 1 medium sized seta and few tiny setae. Meso- and metapleuron, laterotergite and mediotergite bare. Scutellum with 2 strong and several smaller setae.

**Wing.** Fig. 3. Length 4.3 mm. Wing index 2.7. Wings clear, membrane with microtrichia only. Subcosta ending a short distance before base of ta in R. Radial veins, M1, M2, M4 and CuA setose on the dorsal surface, other veins including basal part of CuA bare. Stem of median fork long, about 0.16 times wing length. Length of radial cell 3 times its own width. Vein R5 a little downcurved apically, costa exceeding its tip for 1/3 distance to M1. M4 meets CuA nearly exactly below base of ta. Haltere yellow.

**Legs**. Entirely pale brown. Hind coxae on its posterior ridge with a row of setae. Tibial and tarsal trichiae irregularly arranged. Hind tibiae with 5-7 tiny posteroventral setae in apical half, 6 posterior and 6 anterior setae. Mid tibia with 4 anterior, 4 anterodorsal, 2 posterior and 3 posteroventral setae. Fore tibia with 1 ventral seta. All tibial setae are shorter than tibial width. Mid tibia slightly swollen at its base, with an elongated patch of specialized seta chaetica (Figs 31–32, Table 1). The single setum chaeticum has 2 filamentous branches, subequal in length and width. Hind tibia without apical comb. Tibial spurs 1:2:2, subequal in length. Empodium of normal size, similar to that of *Acomopterella martinovskyi*. Fore basitarsus 1.5 times longer than tibia.

**Abdomen**. All abdominal segments including terminalia brown, with pale hairs. Segments 2-6 long, segment 7 little shorter. Tergite 8 vestigial and completely retracted, sternite 8 reduced in width, partly retracted. **Terminalia.** Figs 26–30. Tergite 9 medium sized, square, together with cerci covering half of the gonocoxites. Gonocoxites ventrally separated, having only a narrow basal sclerotized connection, which possibly represents the remainder of sternite 9. Parameres short, apically rounded. The gonostylus consists of two, completely separated lobes. The dorsal lobus is stout, strongly sclerotized and directed posterad. The ventral lobus is slender, less sclerotized, multicuspidate and directed ventrad. Cerci small, with few apical setae. Hypoproct weakly sclerotized, with two strong apical setae.

Female unknown.

**Table 1.** Details of specialized setae, situated at the upper outer side of tibiae. Abbreviations: Gnor = Gnoristini & Scio = Sciophilini.

Species	Tribe	Figures	Position of tibia		Shape of the single specialized seta					
			mid	hind	simple	complex	foliiform	filamentous	branched	peglike
*Acomopterella yoshiwae*	Gnor	31–32	X			X		X		
*Acomopterella martinovskyi*	Gnor	33–34	X			X		X		
*Coelophthinia thoracica*	Scio	49&55	X		X					
*Docosia diutina*	Gnor	45-48		X	X					X
*Ectrepesthoneura hirta*	Gnor	37-38	X		X?					
*Phthinia humilis*	Scio	50&54	X		X				X	
*Phthinia winnertzi*	Scio	51&57	X		X				X	
*Polylepta guttiventris*	Scio	52&56	X			X	X			
*Polylepta borealis*	Scio	53&58	X			X	X			
*Speolepta leptogaster*	Gnor	35–36	X			X	X			
*Synapha fasciata*	Gnor	43–44	X		X		X			
*Tetragoneura* sp. (FarEast)	Gnor	39–40	X		X		X			
*Tetragoneura* sp. (New Zealand)	Gnor	41–42	X		X		X			

#### Distribution and phenology.

*Acomopterella yoshiwae* sp. n. was collected from an old-growth deciduous / coniferous forest at Mt. Kanmuri on the island of Honshu.

#### Etymology.

The name refers to the origin of this species from Yoshiwa Village, Hiroshima Pref., Honshu, Japan.

#### Holotype.

**male** (on slide), Japan, Honshu, Hiroshima Pref., Yoshiwa Village, Mt. Kanmuri; mature mixed deciduous / coniferous forest; 1000 m; 28 Sep.–22 Oct. 1999; Malaise trap; collector M. Jaschhof (SNSD).

##### Key to species of *Acomopterella*, imagines only

**Table:** 

1	Vein M4 detached at base. With ventral gonocoxal projection only. Striking long cercal setae (Figs 18–20)	*Acomopterella martinovskyi* Ševčík & Chandler
2	M4 meets vein CuA below base of ta. With ventral & dorsal gonocoxal projection	3
3	Gonostylus of 2 separated lobes (Figs 26-27)	*Acomopterella yoshiwae* sp. n.
–	Gonostylus simple, tapered	*Acomopterella fallax* Sherman (Nearctic)

## Discussion

### Inner- and intergeneric relationships

**Figures 14–20.**
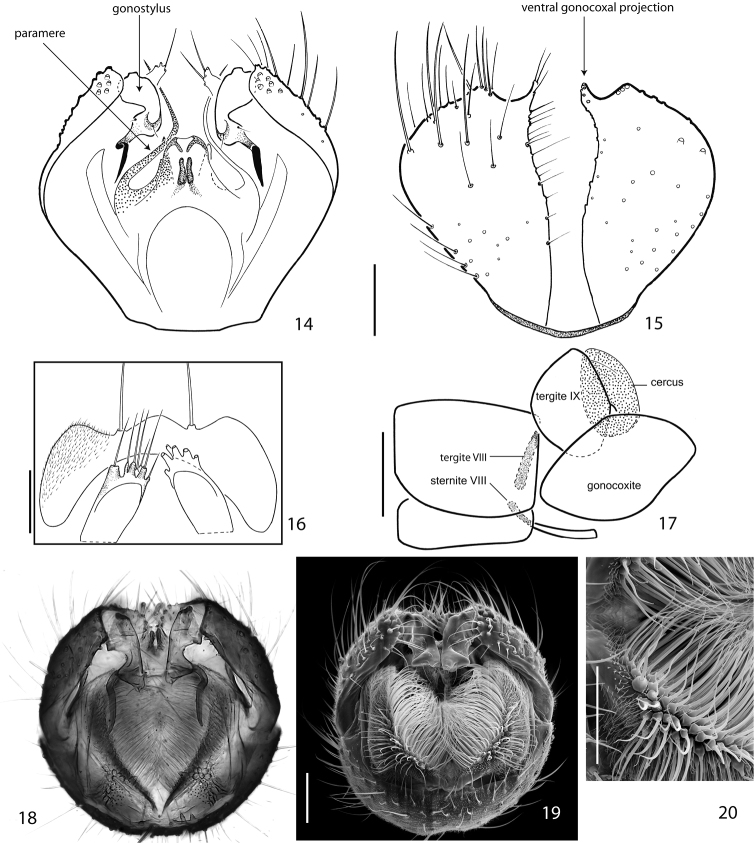
Terminalia of *Acomopterella martinovskyi*, male. **14** Terminalia in dorsal view, tergite IX removed **15** Gonocoxites in ventral view **16** Epiproct and hypoproct, dorsal view **17** Abdominal segments VII & VIII and terminalia in lateral view **18–19** Posterior view of terminalia **20** Cercal setae in detail. Length of scale bar = 100 µm (for **14–15, 18–19**), 50 µm (for **16, 20**), 0,5 mm (for **17**).

The study provides new records of *Acomopterella martinovskyi* from Europe and the first records of this species from eastern Palaearctic localities. This species has a quite low specimen density, thus very few records are known. The new records suggest it is a relict species with 2 distinct allopatric populations: (1) Central & North European – Alpine (2) Far Eastern. The populations are distinct by minimal morphological differences of the male terminalia, i.e. far eastern specimens have the apical half of gonostylus double bent (Fig. 14) and longer than it is in the european specimens. The european specimens are considered to be morphological homogenous, with the apical, tapered half of the gonostylus being shorter and simply bent (Ševčík & Chandler 2008, Figs 3–5). Additionally, the secondspecies was found from only one location on the island of Honshu.

Males of *Acomopterella* have a patch of specialized setae on the outer surface of the mid tibia. The ultrastructure of these setae has been studied using SEM photographs on Palaearctic species of *Acomopterella* and several representative species of other genera of Mycetophilidae. In the herewith studied species these structures are diagnostic on the generic level, e.g. these setae are filamentous in *Acomopterella* and *Ectrepesthoneura*, leaf-like in *Tetragoneura*, leaf-like and slightly curled in *Synapha*, leaf-like and branched in *Speolepta*, filamentous and branched in *Coelophthinia* and *Phthinia*, tapered in *Polylepta*. The present study of the mid tibial organ was restricted to only few genera, mainly to find arguments for the close relationship of *Acomopterella martinovskyi* and *Acomopterella yoshiwae* sp. n. Previous studies did not describe these specialized setae detailed enough. The relative similarity of *Acomopterella* to some other representatives of the clade Gnoristini is discussed below. *Dziedzickia marginata* is different from *Acomopterella*, as stated here, in the relative length of the subcosta, which ends with a rounded bow in R, shortly but distinctly distal from Rs. In addition there are further traits of *Dziedzickia marginata* quite different from *Acomopterella*: mesanepisternum finely hairy along its posterior margin, metepisternum on its entire surface with fine hairs and laterotergite with rather long hairs. Mesanepisternal hairs of medium length, metepisternal hairs short. *Dziedzickia marginata* has a deep sensory pit on the anteromedial side of palpomere 3. Flagellomeres with a touch of polygon-like pattern on its surface. Male abdominal segments 7 and 8 strongly reduced in size, segment 7 partly retracted. Female terminalia rather small, especially cerci. The two segmented cerci are directed posteriad. In comparison to *Acomopterella*, species of *Palaeodocosia*, *Acomoptera*, *Tetragoneura* and *Ectrepesthoneura* have a stouter body and legs and are distinct in their general peculiar construction of male terminalia, including all hitherto studied fossils of these genera (pers. observation, unpublished). *Synapha* is distinct from *Acomopterella* at least by having a conspicuous sensory pit on its 3^rd^ palpomere and male terminalia of simple, i.e. plesiomorphic type. *Archaeboletina* and *Speolepta* are obviously the most closely related genera to *Acomopterella*, especially considering the quite similar wing venation and length of the fore basitarsus. The close relation between *Speolepta* and *Archaeboletina tipuliformis*, from Baltic amber, was already mentioned by Edwards (1940, p. 123). Otherwise, *Archaeboletina* has terminalia of an ancestral, simple structure and tibial setae in distinct rows.

*Speolepta*, *Archaeboletina*, *Dziedzickia marginata*, *Palaeodocosia* and S*ynapha* share all traits that form the clade Gnoristini, as Väisänen (1986) has treated at length, even if the monophyly of Gnoristini is not yet finally established, and thus Gnoristini cannot be reliably defined. But it shows at least, that these genera do not belong to other groups, such as Sciophilinae s. str. (mediotergite setose), Leiinae (weakly delimited by combination of short vein R1 and hind tibial setae distinctly longer than tibial diameter) or Mycetophilinae s. str. (wing microtrichia in definite lines).

*Acomopterella martinovskyi* and*Acomopterella yoshiwae* sp. n. have the anterior transversal vein (ta) quite prolonged and horizontal, together with shortening of R1. This transformation is simply the consequence of wing vein costalization, a general trend in Diptera. It is visible in other Gnoristini as well, e.g. *Tetragoneura* or *Docosia*. There is no doubt that Leiini are a monophyletic group (Jaschhof and Kallweit 2009), though the foreshortened vein R1 may not be seen any longer as an unique synapomorphy of Leiinae.

Despite differences in structure of terminalia, the two Palaearctic *Acomopterella* species under discussion should be assigned to one genus, because details of head, thorax, legs and wings are more similar between them, than to any other species in related genera. On the other hand, presence of two, independently inserted gonostylar lobi on both sides, in comparison to only one lobus, is in fungus gnats usually to be seen as diagnostic on generic level. That is the weak point in the present classification of *Acomopterella yoshiwae*. At the present stage of knowledge, I explain this difference by long-term reproductive isolation of both groups.

Palaearctic species of *Acomopterella* have a prolonged fore basitarsus in common, which is longer than the tibia. *Acomopterella martinovskyi* has the fore basistarsus 1.1 times longer than the tibia, in both populations, eastern and western Palaearctic. In *Acomopterella yoshiwae*sp. n. the fore basitarsus is 1.5 times longer than the tibia. In addition, *Archaeboletina* has the fore basitarsus 1.2 times longer than the tibia. This long fore basitarsus is unique within the clade Gnoristini. Zaitzev (1989) has described *Acomopterella fallax* as having the fore tibia slightly longer than the fore basitarsus.

The subcosta in both species is long, ending immediately in front of the base of the anterior transversal vein. Males have a mid tibial organ on the dorsal basal third of the tibia, females do not. Female cerci are directed ventrad. Considering these features as a complex trait, one may conclude that *Acomopterella yoshiwae* sp. n. and *Acomopterella martinovskyi* are congeneric.

### Modified setae of the legs in selected Mycetophilidae imagines

The legs of Mycetophilidae may possess conspicuously modified setae and other cuticular structures. FORE LEGS: The hitherto best known structure is the “Fore tibial organ” (Fig. 11), which was described in detail by Blaschke-Berthold (1994, p. 50), as mainly consisting of a glandular layer and a comb of setae. This fore tibial organ is an autapomorphy of Sciaroidea. MID LEGS: There is a patch of specialized setae (Figs 31-44, 49-58) found in many species of the Mycetophilidae tribes Sciophilini and Gnoristini (“sensory pit” of Hutson et al. 1980, p.18; “specialized sensory depression” of Vockeroth 1981, p. 226; “tibial sensory groove or pit” by Søli 1997, p. 25). Most recent studies have shown it in species of *Manota* (Manotinae) as well (Jaschhof & Jaschhof 2010, p. 29). A comparative outline of this structure was provided for the first time by Chandler (1980, p. 28), who called it “sensory area” resp. “sensory pit”. The general structure of this area and accessory setae is quite similar among certain species of the same genus (see table A), even though it is frequently absent in species closely related to others bearing it. There are species which exhibit it in both sexes, as in *Coelophthinia* and *Phthinia*, but in the vast majority of the known species, including *Manota*, it is present in males only. The patch of specialized setae on fungus gnat mid tibiae may be an autapomorphy of Mycetophilidae, even though it is most often reduced or not expressed. Its taxonomic value for discrimination of the few studied species is evident, however it is not known well enough for use in higher classification. The specialized tibial setae, recently studied in 13 species in 9 genera of Mycetophilidae, are membranous and transparent. The single seta may be a sensillum chaeticum or glandular chaeta (sensu Francesca Vegliante, pers. comm.). Its possible function remains unclear, pending ongoing histological examination. HIND LEGS: A patch of modified tibial setae is also present in the male of *Docosia diutina* (females are still unknown). It has the same position on the hind tibia (Figs 45-46), as is the case in other taxa on the mid tibia. The single modified seta looks like a clothes peg (Figs 47-48). The area with modified setae is here not sharply delimited, though marked by several densely set tibial setae. This “hind tibial organ” can tentatively be seen as homologous to the “mid tibial organ” in other taxa. This assumption meets the two main criteria of homology: criterion of position and criterion of special structural quality (sensu Storch et. al. 2007, p. 55). It fits the description of “serial homology”, given by Haszprunar (1992). Males of the closely related *Docosia rohaceki* (Ševčík 2006, p. 133) have perhaps the same structure, though roughly described as a “small black spot on hind tibia”. It is absent in the female. It seems to be less certain whether the “hind tibial organ” of *Manota*, described by Jaschhof et al. (2011) is homologous to the mid one because of its location on the tibial tip.

Zaitzev (1989, p. 136) pointed to the similarity between *Acomopterella* and *Austrosynapha*, however his opinion is based on highly variable characters, as length and obliquity of vein ta and setation of laterotergites. The present study has shown that the general structure of the *Acomopterella martinovskyi* eggshell is rather more similar to that of *Austrosynapha* (pers. observation, unpublished) or *Mycomya* (Mazzini et al. 1992) than that of *Speolepta* (Plachter 1981).

Zaitzev’s description of the *Acomopterella* palpomeres (see above) is somewhat unclear, regarding their length. This problem may be solved by studying the type species of the genus, which is beyond the scope of this paper.

## Plates

**Figures 21–25.**
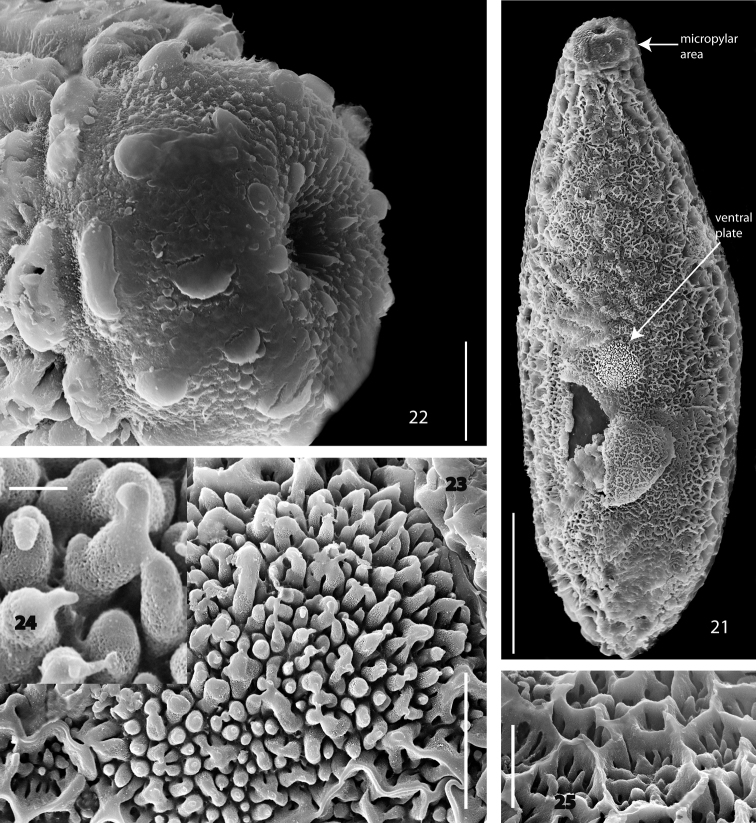
*Acomopterella martinovskyi*, egg. **21** Total view of egg, bottom side **22** Micropylar area **23** Total view of ventral plate **24** Column of ventral plate in detail **25** Hexagonal meshwork of the egg dorsal side. Length of scale bar = 100 µm (for **21**), 10 µm (for **22–23, 25**), 1 µm (for **24**).

**Figures 26–30.**
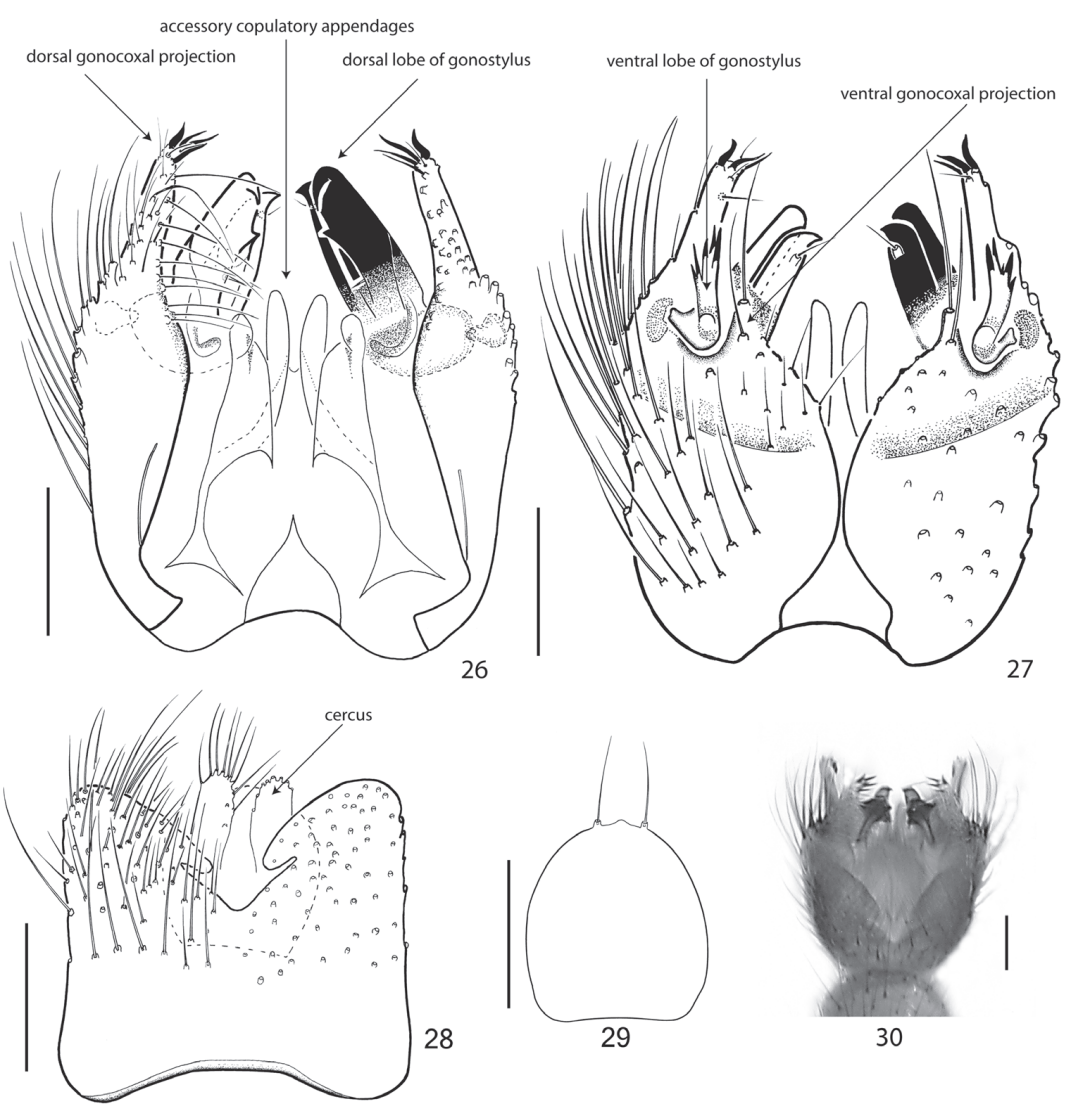
Terminalia of *Acomopterella yoshiwae* sp. n., male. **26** Terminalia in dorsal view, tergite IX removed **27** Terminalia in ventral view **28** Tergite IX, dorsal view **29** Hypoproct, ventral view **30** Terminalia incl. tergite IX in dorsal view. Length of scale bar = 100 µm.

**Figures 31–38.**
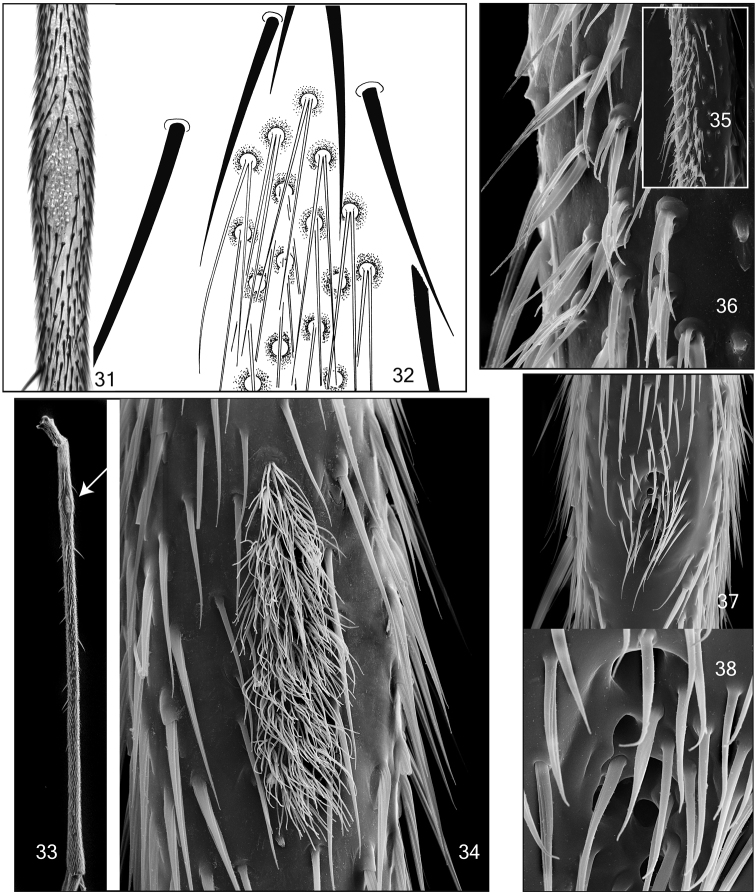
Mid tibial organ. Not on the same scale. **31–32** *Acomopterella yoshiwae* sp. n. **33–34** *Acomopterella martinovskyi* **33** General outer view of mid tibia, the arrowhead points to the tibial organ **35–36** *Speolepta leptogaster* **37–38** *Ectrepesthoneura hirta*.

**Figures 39–48.**
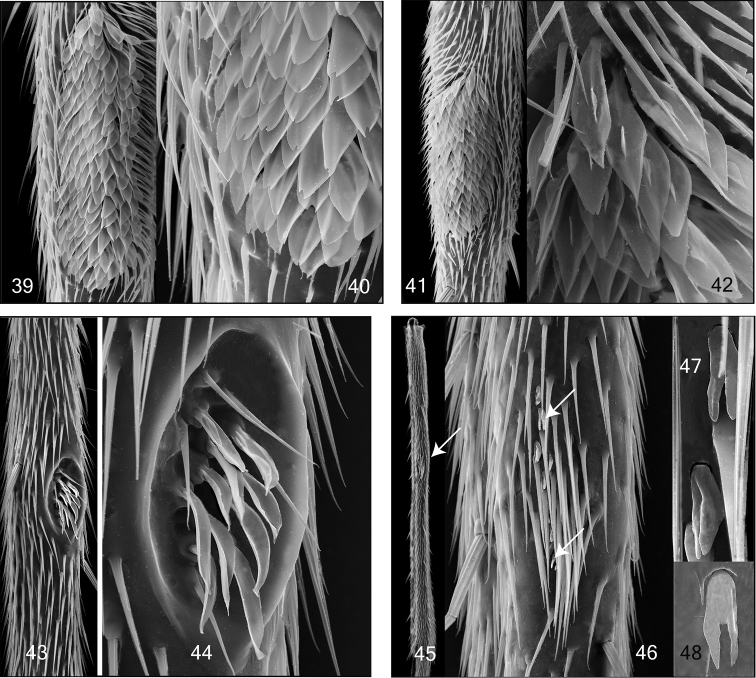
Mid and hind tibial organ. Not on the same scale. **39–40** *Tetragoneura* sp. (New Zealand) mid tibia **41–42** *Tetragoneura* sp. (Far East) mid tibia **43–44** *Synapha fasciata*, mid tibia **45–48** *Docosia diutina*, hind tibia **45** General outer view of hind tibia, the arrowhead points to the tibial organ **46** Tibial organ with densely set setae and and few sensilla chaetica, the arrowheads point to 2 of them **47–48** Sensilla chaetica in detail.

**Figures 49–58.**
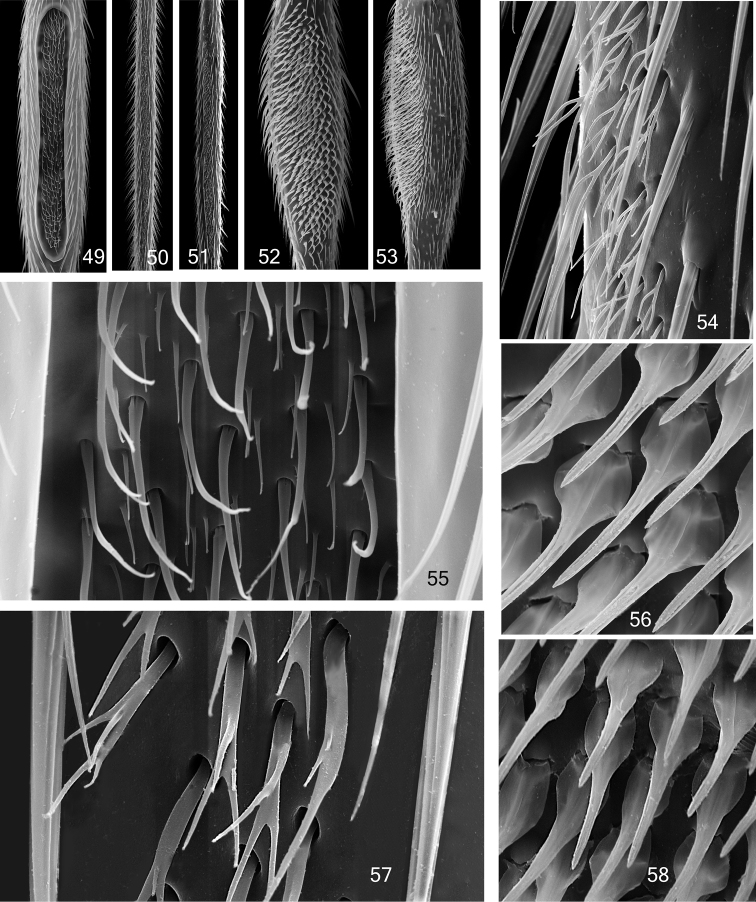
Mid tibial organ. Not on the same scale **49, 55** *Coelophthinia thoracica* **50, 54** *Phthinia humilis* **51, 57** *Phthinia winnertzi* **52, 56** *Polylepta guttiventris* **53, 58** *Polylepta borealis*.

## Supplementary Material

XML Treatment for Acomopterella

XML Treatment for Acomopterella martinovskyi

XML Treatment for Acomopterella yoshiwae
